# A Comprehensive Review of HIV-Associated Tuberculosis: Clinical Challenges and Advances in Management

**DOI:** 10.7759/cureus.68784

**Published:** 2024-09-06

**Authors:** Aniket Patel, Aditya Pundkar, Anshu Agarwal, Charuta Gadkari, Anmol K Nagpal, Nigil Kuttan

**Affiliations:** 1 Emergency Medicine, Jawaharlal Nehru Medical College, Datta Meghe Institute of Higher Education & Research, Wardha, IND; 2 Orthopedics, Jawaharlal Nehru Medical College, Datta Meghe Institute of Higher Education & Research, Wardha, IND

**Keywords:** antiretroviral therapy, clinical management, diagnostic challenges, hiv-tb co-infection, public health strategies, treatment advances

## Abstract

Human immunodeficiency virus (HIV) and tuberculosis (TB) are two of the most pressing global health issues, each contributing significantly to morbidity and mortality worldwide. This review provides a comprehensive analysis of HIV-associated TB (HIV-TB), focusing on the clinical challenges and advancements in its management. HIV-positive individuals are at a heightened risk of developing active TB due to the immunosuppressive effects of the virus, which complicates both diagnosis and treatment. The interplay between these two diseases exacerbates health outcomes, presenting unique challenges related to drug interactions, adherence to treatment regimens, and management of adverse effects. This review explores the current diagnostic approaches, including advances in testing technologies and screening strategies, and examines treatment protocols, highlighting the integration of antiretroviral therapy with TB treatment. Special considerations for managing HIV-TB in various populations, such as children, pregnant women, and the elderly, are discussed. Additionally, the review addresses public health strategies for prevention and the impact of socio-economic and healthcare system factors on disease management. Finally, it highlights recent research innovations and future directions aimed at improving outcomes for individuals with HIV-TB. By synthesizing the latest evidence and clinical practices, this review aims to enhance understanding and guide effective management of this critical co-infection, ultimately contributing to reduced global burden and improved patient care.

## Introduction and background

Human immunodeficiency virus (HIV) and tuberculosis (TB) represent two of the most significant global health challenges, each contributing to substantial morbidity and mortality worldwide. TB, caused by the bacterium Mycobacterium tuberculosis, continues to be one of the leading infectious diseases [1]. According to the World Health Organization (WHO), in 2022, approximately 10.6 million people were diagnosed with TB globally, with around 1.6 million cases attributed to co-infection with HIV [2]. HIV, with an estimated 38 million people living with the virus as of 2022, weakens the immune system by targeting CD4+ T cells. This immunosuppression increases susceptibility to opportunistic infections, including TB, creating a particularly challenging scenario for affected individuals [3].

The intersection of HIV and TB presents a complex public health issue due to their mutually exacerbating effects. HIV-positive individuals face a significantly higher risk of developing active TB compared to those who are HIV-negative [4]. This elevated risk is primarily due to the immunocompromised state induced by HIV, which undermines the body's ability to control latent TB infections and mount an effective response to active TB. As a result, the dual burden of these diseases complicates diagnosis and treatment, leading to more severe health outcomes and an increased risk of mortality. Managing HIV-associated TB (HIV-TB) requires addressing the intricacies of drug interactions, side effects, and adherence to both TB and antiretroviral therapies [5].

Studying HIV-TB is crucial because of its profound impact on public health and healthcare systems. The dual challenges of diagnosing and treating these co-infections necessitate a deeper understanding of their interplay to improve patient outcomes [6]. Effective management strategies and healthcare delivery can be significantly enhanced by elucidating the interactions between HIV and TB. This review aims to provide a comprehensive overview of HIV-TB, exploring the clinical challenges and recent advances in management [4]. By examining the current knowledge, treatment strategies, and public health implications, the review seeks to contribute to improved clinical practices and policies, ultimately aiming to reduce the global burden of HIV-TB.

## Review

Pathophysiology

Interaction Between HIV and Mycobacterium Tuberculosis

**Figure 1:**
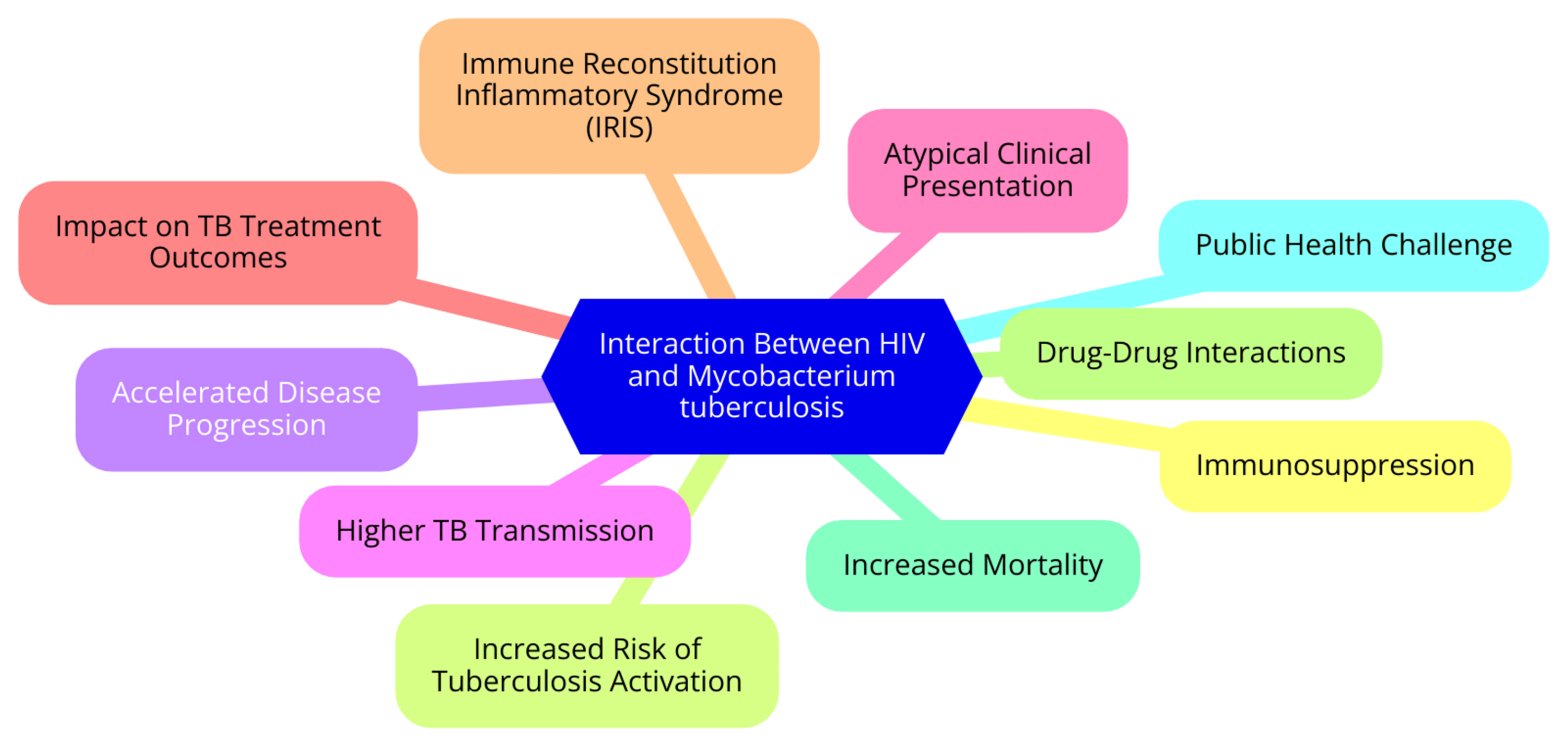
The interaction between HIV and Mycobacterium tuberculosis Image Credit: Dr Aniket Patel TB: Tuberculosis

The interaction between HIV and Mycobacterium tuberculosis (M. tuberculosis) significantly impacts the pathophysiology of HIV-TB. HIV primarily targets CD4+ T cells, causing their gradual depletion and profound immunosuppression [7]. This depletion weakens the immune system’s ability to respond to infections, including TB effectively. As CD4+ T cell counts decline, the body becomes increasingly vulnerable to both initial infection with M. tuberculosis and the reactivation of latent TB [7]. Additionally, HIV alters the functionality of other immune cells, such as macrophages and dendritic cells, which are vital for recognizing and controlling TB infections. Specifically, HIV-infected macrophages show impaired phagocytic activity and a reduced capacity to kill M. tuberculosis, allowing the pathogen to survive and replicate [8]. The combined effects of HIV and M. tuberculosis create a synergistic relationship that worsens disease progression. Individuals co-infected with HIV and TB face a substantially higher risk of developing active TB, with studies showing that the likelihood of reactivating latent TB infections is significantly increased [9]. This dynamic sets off a vicious cycle in which HIV infection not only increases susceptibility to TB but also complicates the immune response to TB, potentially speeding up the progression from HIV infection to AIDS. As a result, dual infection leads to higher morbidity and mortality rates, highlighting the urgent need for effective management strategies [9]. The interaction between HIV and M. tuberculosis is illustrated in Figure 1.

Co-infection Effects

HIV co-infection exacerbates TB progression through several mechanisms. The immunocompromised state of HIV-infected individuals permits rapid bacterial replication and dissemination throughout the body. This accelerated progression often presents as more severe forms of TB, such as disseminated TB or extrapulmonary TB, which are associated with poorer clinical outcomes [10]. Additionally, the inflammatory response triggered by TB can lead to immune reconstitution inflammatory syndrome (IRIS) when antiretroviral therapy (ART) is initiated. In IRIS, the recovering immune system mounts an excessive response to the TB infection, increasing morbidity and complicating the clinical management of both infections [10]. The immune system's interactions during co-infection are intricate and multifaceted. While the depletion of CD4+ T cells is a hallmark of HIV infection, the virus also impairs the function of other immune cells, including CD8+ T cells and natural killer cells, which are crucial for controlling TB [11]. This dysregulation further weakens the immune response, allowing TB to progress more quickly and severely than in immunocompetent individuals. Moreover, the inflammatory cytokines produced in response to TB can adversely affect the immune response to HIV, creating a harmful feedback loop that worsens both infections [11].

Clinical Presentation

The most common symptoms of HIV-TB include a cough (often with sputum or blood) lasting more than three weeks, fever, night sweats, weight loss, and fatigue. However, up to 50% of people living with HIV who are on ART may be asymptomatic for TB [12]. Additionally, HIV-infected patients with TB are more likely to present with atypical chest radiograph findings, such as infiltrates in the lower lung zones, lymphadenopathy, and pleural effusions, compared to those who are HIV-uninfected [13]. Diagnosing TB in HIV patients is challenging due to the lower incidence of cavitary disease and higher rates of smear-negative disease, which makes sputum microscopy less sensitive. There is also a greater risk of extrapulmonary TB, necessitating diagnostic tests beyond sputum analysis, as well as the similarity of symptoms to other opportunistic infections in advanced HIV [14]. The risk of extrapulmonary TB increases with decreasing CD4 cell counts in HIV patients. Common extrapulmonary sites include the lymph nodes (especially peripheral nodes), pleura, central nervous system, pericardium, and bones and joints. Disseminated TB, involving multiple sites, is also more common in advanced stages of HIV. Diagnosing extrapulmonary TB is frequently done without bacteriological confirmation [15]. New diagnostic tools, such as Xpert Ultra and lateral flow urine lipoarabinomannan assays (LF-LAM), offer improved sensitivity for diagnosing TB in HIV patients, particularly in those with advanced immunosuppression. Despite these advancements, a significant portion of HIV-TB cases continue to be diagnosed clinically without microbiological confirmation [16]. The clinical presentation of HIV-TB is detailed in Table 1.

**Table 1:** Clinical presentation of HIV-associated tuberculosis TB: Tuberculosis

Clinical Aspect	HIV-Associated Tuberculosis	Characteristics/Details
Symptom Variability [17]	Increased variability compared to HIV-negative TB	Symptoms may range from typical to atypical and may include non-specific symptoms.
Pulmonary TB [17]	Common presentation in HIV-positive individuals	Symptoms: chronic cough, hemoptysis, night sweats, weight loss, and fever.
Extrapulmonary TB [17]	More frequent in HIV-positive individuals	It can affect lymph nodes, pleura, bones, kidneys, abdomen, and central nervous system.
Subclinical or Atypical [17]	Possible due to advanced immunosuppression	May present with mild or no symptoms; difficulty in diagnosis.
Diagnostic Challenges [17]	Higher risk of atypical presentations	Standard tests like chest X-rays and sputum smears may be less reliable; more sensitive diagnostic methods are needed.
TB Immune Reconstitution Syndrome (IRIS) [17]	Potential complications during ART initiation	Exacerbation of TB symptoms or development of new symptoms after starting ART.

Diagnostic approaches

The Centers for Disease Control and Prevention (CDC) recommends routine HIV screening for all patients aged 13 to 64 as part of standard healthcare practices. Patients with risk factors for HIV should undergo more frequent testing, such as annually or every 3-6 months. Opt-out screening, where patients are informed that an HIV test will be conducted unless they decline, has been effective in reducing stigma and promoting earlier diagnosis [18]. Diagnostic tests for TB in HIV patients include sputum smear microscopy, mycobacterial culture, chest X-ray, and nucleic acid amplification tests (NAATs). Sputum smear microscopy has limited sensitivity in HIV-positive individuals due to a lower bacillary load and a higher prevalence of extrapulmonary TB [19]. Mycobacterial culture is considered the gold standard but is restricted by lengthy turnaround times and the need for specialized laboratory facilities. Chest X-ray can be useful but lacks specificity, especially in patients with advanced HIV. NAATs, such as Xpert MTB/RIF, provide rapid and sensitive detection of TB and drug resistance but may have reduced performance in diagnosing HIV-TB [20]. Newer technologies, such as Xpert Ultra-a NAAT with enhanced sensitivity compared to Xpert MTB/RIF, particularly for HIV-TB have been developed. Interferon-gamma release assays (IGRAs) can help differentiate latent from active TB but show variable performance in HIV-positive individuals. Urine lipoarabinomannan (LAM) assays can quickly diagnose TB in HIV patients with advanced immunosuppression and have been integrated into WHO guidelines [21]. Despite these advancements in diagnostics, a significant number of HIV-TB cases remain undiagnosed or are diagnosed late, leading to high morbidity and mortality. Ongoing research is focused on developing more sensitive and specific tests that can be used at the point of care, reducing diagnostic delays and improving outcomes for this vulnerable population [22]. Diagnostic approaches for HIV-TB are shown in Table 2.

**Table 2:** Diagnostic approaches for HIV-associated tuberculosis

Diagnostic Approach	Description	Advantages	Limitations
Sputum Smear Microscopy [23]	Examination of sputum samples under a microscope for acid-fast bacilli (AFB).	Simple, cost-effective, rapid results.	Lower sensitivity in HIV-positive patients; not effective for extrapulmonary TB.
Culture [23]	Culturing sputum samples to grow Mycobacterium tuberculosis for definitive diagnosis.	Highly sensitive and specific; detects drug resistance.	Time-consuming (weeks to months); requires specialized facilities.
Chest X-ray [23]	Imaging of the chest to identify lung abnormalities associated with TB.	Non-invasive; helps detect pulmonary lesions.	Limited specificity; cannot confirm TB without additional tests.
GeneXpert (Xpert MTB/RIF) [23]	Molecular test that detects TB DNA and rifampicin resistance.	Rapid results; high sensitivity and specificity.	It requires specialized equipment; the cost can be high.
Interferon-Gamma Release Assays (IGRAs) [23]	Blood tests measure the immune response to TB-specific antigens.	High specificity; useful for diagnosing latent TB.	Limited utility in active TB diagnosis; not widely available.
Tuberculin Skin Test (TST) [23]	Intradermal injection of purified protein derivative (PPD) to assess immune response.	Inexpensive; widely used.	False positives/negatives in HIV patients; does not distinguish between latent and active TB.
Sputum PCR [23]	Polymerase chain reaction test to detect TB DNA in sputum samples.	Highly sensitive; detects TB even in small quantities.	High cost; requires specialized facilities.

Management and treatment

The management of HIV-TB necessitates a comprehensive approach that combines effective TB treatment with ART. The standard treatment regimen for drug-susceptible TB typically involves a six-month course of four first-line antitubercular drugs: isoniazid (INH), rifampicin (RIF), pyrazinamide, and ethambutol (EMB). In HIV-positive patients, particularly those with low CD4 counts or severe disease, the treatment duration may be extended to nine months, especially in cases of extrapulmonary TB or TB meningitis. Close monitoring for drug efficacy and side effects is essential due to the potential for increased toxicity in HIV patients [23]. Managing drug-resistant TB (DR-TB) in HIV patients presents additional challenges. Treatment regimens for DR-TB may include second-line drugs such as fluoroquinolones (e.g., moxifloxacin) and injectable agents (e.g., amikacin), often requiring longer treatment durations, sometimes up to 18-24 months. Given the complexity of DR-TB, individualized treatment plans based on drug susceptibility testing are crucial. Coordination with ART is necessary to avoid drug interactions and optimize treatment effectiveness [24]. The timing of ART initiation in patients with HIV-TB is critical. Current guidelines recommend starting ART within 2-8 weeks of initiating TB treatment, particularly for patients with CD4 counts below 350 cells/mm³, to reduce morbidity and mortality. In some cases, especially for patients with severe TB or those at high risk for IRIS, ART may be delayed until the patient has stabilized on TB treatment [25]. Drug interactions between ART and anti-TB medications can complicate treatment. RIF, a potent inducer of cytochrome P450 enzymes, can reduce the efficacy of certain ART medications, particularly non-nucleoside reverse transcriptase inhibitors (NNRTIs) like efavirenz and nevirapine. Using integrase strand transfer inhibitors (INSTIs) such as dolutegravir or bictegravir, which have fewer interactions with RIF, can be considered. Closely monitoring drug levels and patient response is crucial to effectively manage potential interactions [25].

Both TB and ART medications can cause significant side effects, including hepatotoxicity (from isoniazid and RIF), gastrointestinal disturbances, and neurological effects (from isoniazid). Regular monitoring of liver function tests, complete blood counts, and renal function is necessary to detect and manage adverse effects early. Educating patients about potential side effects and when to seek medical attention is vital for timely intervention [26]. Effective management of drug interactions is crucial for optimizing treatment. Healthcare providers should regularly review all medications a patient is taking to identify potential interactions. Adjusting the dosages of ART or TB medications may be necessary based on the patient’s response and side effects. In complex cases, therapeutic drug monitoring (TDM) can help optimize drug levels and ensure therapeutic efficacy while minimizing toxicity [27]. The management of HIV-TB requires a comprehensive and integrated approach. By adhering to established treatment protocols, carefully timing ART initiation, and proactively managing adverse effects and drug interactions, healthcare providers can significantly improve outcomes for patients facing this dual epidemic. Ongoing research and adaptation of treatment strategies will be essential in addressing the evolving challenges of HIV-TB coinfection [27]. The management and treatment strategies for HIV-TB are summarized in Table 3.

**Table 3:** The management and treatment strategies for HIV-associated tuberculosis

Aspect	Details
Treatment Protocols [28]	- Standard TB Regimen: Usually includes a combination of rifampicin, isoniazid, pyrazinamide, and ethambutol (RIPE). - Modifications for HIV: Adjustments may be needed due to drug interactions with antiretroviral therapy (ART).
Antiretroviral Therapy (ART) [28]	- Timing of Initiation: ART should generally be started as soon as possible in patients with HIV-associated TB, preferably within the first eight weeks of TB treatment. - Drug Interactions: Careful management of potential interactions between ART and TB medications is crucial.
Drug Interactions [28]	- Rifampicin and ART: Rifampicin can reduce the effectiveness of certain ART drugs (e.g., protease inhibitors and non-nucleoside reverse transcriptase inhibitors). - Alternatives: Rifabutin or dose adjustments for ART may be necessary.
Management of Adverse Effects [28]	- TB Medication Side Effects: Monitor for common side effects such as hepatotoxicity, peripheral neuropathy, and hypersensitivity reactions. - ART Side Effects: Manage potential side effects of ART, including gastrointestinal issues, metabolic disturbances, and immune reconstitution inflammatory syndrome (IRIS).
Special Populations [28]	- Pediatric Patients: Use age-appropriate formulations and monitor for growth and development issues. - Pregnant Women: Manage TB and HIV with considerations for fetal safety and potential drug interactions. - Elderly Patients: Adjust dosages and monitor for age-related complications.
Monitoring and Follow-Up [28]	- Regular Monitoring: Frequent clinical and laboratory assessments to track treatment response, side effects, and adherence. - Drug Resistance Testing: Conduct regular testing for drug resistance in cases of treatment failure or non-adherence.
Preventive Measures [28]	- TB Prophylaxis: Consider isoniazid preventive therapy for HIV-positive individuals with latent TB infection. - Vaccination: Evaluate the use of BCG vaccination in high-risk populations.

Special populations and considerations

Pediatric Patients

Children living with HIV face significant challenges in diagnosing and treating TB. The clinical presentation of TB in pediatric patients is often atypical, which can lead to delays in diagnosis. Traditional diagnostic methods, such as sputum smear microscopy, are less effective in young children, making it necessary to use more sensitive techniques like nucleic acid amplification tests. Moreover, the overlap of symptoms from other HIV-related lung diseases further complicates the diagnostic process, underscoring the need for healthcare providers to maintain a high index of suspicion for TB in this vulnerable population [28]. Treatment regimens for HIV-TB in children must be carefully customized due to the potential for drug-drug interactions between ART and anti-TB medications. Current guidelines recommend a six-month regimen that includes RIF. Still, the optimal timing for initiating ART during TB treatment remains a matter of debate, particularly in children with advanced immunosuppression [29]. There is also considerable concern about managing post-tuberculosis lung disease, which can be more severe in children with HIV. Overall, an integrated approach that addresses the specific needs of pediatric patients is essential for effective management [30].

Pregnant Women

Managing HIV-TB during pregnancy involves specific challenges that require balancing the treatment of both conditions while minimizing risks to both mother and fetus. Pregnant women with HIV face an increased risk of TB, which can progress rapidly during pregnancy. ART is essential for improving maternal health and reducing the risk of vertical transmission of HIV; however, the selection of medications must be carefully considered to avoid teratogenic effects [30]. When treating TB in pregnant women, standard protocols are generally followed, but adjustments are necessary to ensure safety for both mother and child. First-line anti-TB medications such as isoniazid, RIF, and EMB are usually considered safe during pregnancy. Nevertheless, the timing of ART initiation must be carefully managed to balance the risks of maternal immunosuppression against potential adverse effects on fetal development. Close monitoring and a multidisciplinary approach are crucial to optimizing outcomes for both the mother and the child, ensuring effective management of both HIV and TB [31].

Elderly Patients

Elderly patients with HIV-TB encounter unique challenges due to age-related physiological changes, comorbidities, and polypharmacy. Diagnosing TB in older adults can be particularly difficult because of atypical presentations and the presence of other respiratory conditions that may obscure TB symptoms. Consequently, heightened clinical suspicion and more comprehensive diagnostic evaluations, including imaging and microbiological tests, are necessary for timely and accurate diagnosis [31]. Treatment regimens for elderly patients must be carefully managed to account for potential drug interactions and the increased risk of adverse effects from both TB and HIV medications. Additionally, the risk of IRIS upon initiating ART is a concern in this population, requiring careful timing and monitoring. A tailored approach that addresses the specific health profiles and needs of elderly patients is crucial for the effective management of HIV-TB, ensuring appropriate care while minimizing risks [32]. Special populations and considerations in managing HIV-TB are detailed in Table 4.

**Table 4:** Special populations and considerations in managing HIV-associated tuberculosis TB: Tuberculosis

Population	Considerations	Challenges	Management Strategies
Pediatric Patients [33]	Differences in clinical presentation compared to adults	Difficulty in diagnosing due to atypical symptoms	Use of age-appropriate diagnostic tests; close monitoring of treatment response; tailored dosing of medications.
Pregnant Women [33]	Risks of both TB and HIV to maternal and fetal health	Potential teratogenic effects of TB drugs; drug interactions with ART	Coordination of TB and ART treatment to minimize risks; use of safer medications; regular monitoring.
Elderly Patients [33]	Increased likelihood of comorbidities and polypharmacy	Potential for drug interactions and altered drug metabolism	Adjustments in drug dosing; careful monitoring for side effects and drug interactions.
Immunocompromised [33]	Enhanced susceptibility to severe forms of TB	Complications with TB treatment and ART adherence	Strengthening of ART adherence; intensive TB monitoring and treatment adjustments.
People with Drug-Resistant TB [33]	Complex treatment regimens and increased risk of treatment failure	Limited options for effective therapy	Use of second-line TB drugs; individualized treatment plans; regular follow-up and monitoring.
Patients with Co-morbid Conditions [33]	Impact of other conditions (e.g., diabetes, hepatitis) on TB and HIV management	Potential exacerbation of disease interactions and treatment complications	Comprehensive management of co-morbidities; coordinated care approach to address multiple health issues.

Public health and prevention strategies

TB preventive treatment (TPT) is essential for reducing the risk of active TB disease in individuals living with HIV. The most commonly used regimen is 6-12 months of isoniazid preventive therapy (IPT), which has been shown to decrease the risk of TB by 40-60%. Despite its efficacy, IPT uptake is still limited. The WHO conditionally recommends longer courses of at least 36 months in high TB transmission settings [34]. Shorter TPT regimens, such as those using rifapentine, can enhance availability and facilitate broader implementation. The WHIP3TB trial found that a 1-month regimen of daily rifapentine plus isoniazid was non-inferior to a 3-month regimen of weekly rifapentine plus isoniazid for TB prevention in people living with HIV [34]. The BCG vaccine, while not highly effective in preventing pulmonary TB in adults-including those with HIV-may still be beneficial for reducing the risk of severe forms of TB, such as miliary TB and TB meningitis, in HIV-positive infants and young children [35]. New TB vaccine candidates are undergoing clinical trials, but their efficacy in HIV-positive populations has yet to be established. The M72/AS01E vaccine candidate demonstrated 54% efficacy in preventing progression to pulmonary TB disease in HIV-negative adults with latent TB infection [36]. Improving access to TB and HIV services is crucial for addressing health disparities. Decentralized and integrated TB and HIV care models in high-burden settings can promote earlier diagnosis and treatment. Strategies such as provider-initiated HIV testing and counseling in TB clinics, along with intensified TB case finding among individuals living with HIV, are important [37]. Addressing social determinants of health, including poverty and stigma, is also vital. Interventions targeting vulnerable populations, such as children, pregnant women, and key populations at risk for both TB and HIV, are necessary [38]. Public health and prevention strategies for HIV-TB are shown in Table 5.

**Table 5:** Public health and prevention strategies for HIV-associated tuberculosis TB: Tuberculosis

Strategy	Description	Key Considerations
Routine Screening [39]	Regular screening for TB in HIV-positive individuals to identify latent TB infections and prevent progression to active TB.	Implementation in high-risk populations; cost-effectiveness.
Preventive Therapy [39]	Use of prophylactic treatments such as isoniazid preventive therapy (IPT) to reduce the risk of developing active TB in those with latent TB.	Adherence to therapy; monitoring for potential side effects.
Vaccination [39]	BCG (Bacillus Calmette-Guérin) vaccination for TB prevention, particularly in children and high-risk populations.	Efficacy in HIV-positive individuals; potential for adverse effects.
Integration of Services [39]	Coordinating TB and HIV care services to streamline treatment, improve patient outcomes, and enhance adherence to both TB and antiretroviral therapies.	Need for multidisciplinary approach; logistical and resource considerations.
Education and Awareness [39]	Public health campaigns to educate communities about the risks of HIV and TB, and the importance of screening and preventive measures.	Tailoring messages to specific populations; addressing stigma.
Addressing Socioeconomic Barriers [39]	Implementing interventions to reduce poverty and improve access to healthcare services, which can impact TB and HIV management.	Focus on reducing health disparities and improving access.
Monitoring and Evaluation [39]	Ongoing assessment of TB and HIV prevention programs to ensure effectiveness, identify gaps, and guide improvements.	Importance of data collection and analysis; adapting strategies based on findings.

Challenges and barriers

The management of HIV-TB is fraught with numerous challenges and barriers that impede effective care and treatment. These challenges can be broadly categorized into healthcare system issues and socioeconomic and cultural barriers [39]. One major barrier is the resource limitations and infrastructure challenges faced by healthcare systems, particularly in low- and middle-income countries. Many of these regions struggle with fragmented services, where HIV and TB care are delivered in separate healthcare facilities. This separation complicates patient management, requiring individuals to navigate multiple settings, which often leads to delays in diagnosis and treatment. Furthermore, the integration of TB and HIV services is frequently suboptimal, with some settings implementing only partial integration models. This results in inefficiencies and poor patient outcomes [40]. Additionally, there is a shortage of adequately trained healthcare personnel capable of managing both HIV and TB effectively. This shortage can result in inadequate supervision and support for integrated care models, exacerbating the difficulties faced by patients. Infrastructure deficiencies, such as poorly designed healthcare facilities and inadequate infection control measures, also hinder the delivery of effective care. These systemic issues create significant barriers to the timely and effective management of HIV-TB co-infection [41]. Socioeconomic factors and cultural attitudes further impact the management of HIV-TB co-infection. High levels of poverty can severely limit access to healthcare services, as individuals may prioritize basic needs over seeking medical care. Economic constraints also affect adherence to treatment regimens, particularly for those requiring long-term therapy. Patients may struggle to afford transportation to healthcare facilities or the costs associated with medications, leading to interruptions in treatment [42]. Stigma associated with both HIV and TB remains a major obstacle to accessing care. Many individuals avoid seeking treatment due to fear of discrimination or social ostracism, resulting in delays in diagnosis and treatment initiation. Cultural beliefs and misinformation about both diseases often perpetuate this stigma. Additionally, a lack of health education regarding HIV and TB can impede effective management. Misunderstandings about the diseases, their transmission, and treatment options can lead to poor health-seeking behaviors and low adherence to treatment regimens. Educational initiatives are essential for improving knowledge and reducing stigma in affected communities [43]. The challenges and barriers in managing HIV-TB are summarized in Table 6.

**Table 6:** The challenges and barriers in managing HIV-associated tuberculosis TB: Tuberculosis

Category	Challenges and Barriers	Description
Diagnostic Difficulties [44]	Overlapping Symptoms	Symptoms of HIV-associated TB can overlap with other conditions, complicating accurate diagnosis.
	Limited Access to Diagnostic Tools	Inadequate access to advanced diagnostic technologies, especially in low-resource settings.
	False Negatives in TB Tests	Lower sensitivity of some TB tests in HIV-positive individuals, leading to missed diagnoses.
Treatment Complexity [44]	Drug Interactions	Potential interactions between anti-TB medications and antiretroviral drugs, affecting efficacy and safety.
	Adherence Issues	Difficulty in maintaining adherence to both TB and HIV treatment regimens due to complex schedules and side effects.
	Management of Adverse Effects	Handling the side effects of combined therapies, including drug toxicity and immune reconstitution syndrome.
Healthcare System Constraints [44]	Resource Limitations	Insufficient healthcare resources, including medication availability and healthcare infrastructure.
	Stigma and Discrimination	Social stigma related to HIV and TB can hinder access to care and adherence to treatment.
	Fragmented Care Systems	Lack of integration between HIV and TB care services, leading to suboptimal management.
Public Health Challenges [44]	Inadequate Screening Programs	Insufficient screening and early detection programs, especially in high-risk populations.
	Socioeconomic Factors	Poverty and lack of education contribute to increased risk and poor management of both diseases.
	Policy and Funding Gaps	Limited funding and policy support for comprehensive HIV-TB co-infection programs.

Advances and future directions

The TB drug pipeline has seen considerable expansion in recent years, with 26 drugs currently in Phase I, II, or III clinical trials as of September 2022. This includes 17 new chemical entities, two drugs with accelerated regulatory approval, one drug recently approved by the FDA under the limited population pathway, and six repurposed drugs. Various combination regimens, incorporating either new or repurposed drugs, as well as host-directed therapies, are also being evaluated in late-stage trials [45]. Notable developments include bedaquiline and delamanid, which have received accelerated approval for multidrug-resistant TB (MDR-TB), and pretomanid, a new drug recently approved by the FDA under the limited population pathway for antibacterial and antifungal drugs [46]. Similarly, the TB diagnostic pipeline has expanded significantly, with numerous new molecular tests, biomarkers, and technologies under development. This includes molecular tests for detecting TB disease and drug resistance, interferon gamma release assays (IGRAs) for diagnosing TB infection, biomarker-based assays for detecting TB disease, computer-aided detection systems for TB screening using digital chest radiography, and aerosol-capture technologies for TB disease detection [47]. In 2022, the WHO evaluated and recommended three new antigen-based skin tests for TB infection, which have shown better specificity than the traditional tuberculin skin test [48]. The WHO continues to provide policy guidance and recommendations to support the scaling up of new TB diagnostics, drugs, and treatment regimens. Recent updates include recommendations for new antigen-based skin tests for TB infection diagnosis, guidance on incorporating bedaquiline and delamanid into MDR-TB treatment regimens, and advice on the timing of antiretroviral therapy initiation in HIV-TB. Specifically, ART should commence within 2-8 weeks of starting TB treatment for patients with CD4 counts below 50 cells/μL [49]. Despite these advancements, significant gaps remain in TB research and innovation. These include insufficient funding, with only $915 million available in 2020 compared to the global target of $2 billion per year. There is also a need for more operational research to evaluate the real-world usability and impact of new tools, a lack of data on the performance of new diagnostics and drugs in key populations such as children and those with advanced HIV, and the urgent need for major technological breakthroughs, such as an effective pre- and post-exposure TB vaccine, to meet the ambitious End TB Strategy targets. Addressing these gaps will require sustained political will, increased funding, innovative partnerships, and a sense of urgency from all stakeholders to accelerate the development and implementation of new TB tools [50]. Emerging therapies and drug development for HIV-TB are summarized in Table 7.

**Table 7:** Emerging therapies and drug development for HIV-associated tuberculosis TB: Tuberculosis

Therapy/Drug	Description	Mechanism of Action	Current Status	Key Considerations
Bedaquiline [51]	Novel diarylquinoline with potent anti-TB activity.	Inhibits ATP synthase, disrupting energy production in M. tuberculosis.	Approved for MDR-TB; ongoing research for use in HIV-TB co-infection.	Potential for drug interactions with ART; need for close monitoring.
Delamanid [52]	Nitroimidazole derivative with anti-TB properties.	Inhibits mycolic acid biosynthesis, affecting cell wall integrity.	Approved for MDR-TB; research ongoing for broader use.	Risk of QT prolongation; interactions with HIV medications.
Pretomanid [52]	Part of the BPaL regimen (Bedaquiline, Pretomanid, Linezolid).	Inhibits mycobacterial cell wall synthesis and DNA replication.	Approved for MDR-TB; studies ongoing for HIV co-infection.	High cost; drug interactions with ART.
Imipenem-Cilastatin [53]	Carbapenem antibiotic used in combination with other TB drugs.	Inhibits bacterial cell wall synthesis.	Under investigation for MDR-TB; potential benefits in HIV-TB co-infection.	Limited data in HIV-TB; potential for resistance.
Moxifloxacin [53]	Fluoroquinolone with activity against M. tuberculosis.	Inhibits DNA gyrase and topoisomerase IV, crucial for DNA replication.	Approved for use in MDR-TB; research ongoing for HIV-TB co-infection.	Risk of QT prolongation; interactions with ART.
Newer Macrolides (e.g., Azithromycin) [54]	Macrolides with potential activity against TB.	Inhibits bacterial protein synthesis.	Emerging evidence in clinical trials for HIV-TB co-infection.	Limited data; potential interactions with ART.

## Conclusions

In conclusion, the intersection of HIV and TB presents a formidable public health challenge that demands a nuanced and multifaceted approach to management. The synergistic effects of these two diseases exacerbate the complexity of diagnosis and treatment, significantly impacting patient outcomes and healthcare systems worldwide. Effective management of HIV-associated TB requires a comprehensive understanding of the interactions between these conditions, as well as an integration of advanced diagnostic techniques and treatment strategies. Recent advances in research and clinical practice promise to improve the management of this co-infection, yet considerable work remains to address ongoing challenges and disparities. By focusing on enhanced diagnostic tools, innovative treatment regimens, and robust public health strategies, we can make significant strides toward mitigating the burden of HIV-associated TB. This review underscores the importance of continued research and policy development to refine our approaches and ultimately reduce the global impact of this critical co-infection.

## References

[1] Bloom BR, Atun R, Cohen T (2017). Tuberculosis. Major Infectious Diseases.

[2] (2024). Tuberculosis (TB). https://www.who.int/news-room/fact-sheets/detail/tuberculosis.

[3] Waymack JR, Sundareshan V (2024). Acquired immune deficiency syndrome. StatPearls [Internet].

[4] Bhatt A, Quazi Syed Z, Singh H (2023). Converging epidemics: a narrative review of tuberculosis (TB) and human immunodeficiency virus (HIV) coinfection. Cureus.

[5] Walker NF, Meintjes G, Wilkinson RJ (2013). HIV-1 and the immune response to TB. Future Virol.

[6] Bruchfeld J, Correia-Neves M, Källenius G (2015). Tuberculosis and HIV coinfection. Cold Spring Harb Perspect Med.

[7] Geldmacher C, Schuetz A, Ngwenyama N (2008). Early depletion of Mycobacterium tuberculosis-specific T helper 1 cell responses after HIV-1 infection. J Infect Dis.

[8] Abrahem R, Chiang E, Haquang J, Nham A, Ting YS, Venketaraman V (2020). The role of dendritic cells in TB and HIV infection. J Clin Med.

[9] Shankar EM, Vignesh R, Ellegård R (2014). HIV-Mycobacterium tuberculosis co-infection: a 'danger-couple model' of disease pathogenesis. Pathog Dis.

[10] Azevedo-Pereira JM, Pires D, Calado M, Mandal M, Santos-Costa Q, Anes E (2023). HIV/Mtb co-Infection: from the amplification of disease pathogenesis to an "emerging syndemic". Microorganisms.

[11] Février M, Dorgham K, Rebollo A (2011). CD4+ T cell depletion in human immunodeficiency virus (HIV) infection: role of apoptosis. Viruses.

[12] Hamada Y, Getahun H, Tadesse BT, Ford N (2021). HIV-associated tuberculosis. Int J STD AIDS.

[13] San KE, Muhamad M (2001). Pulmonary tuberculosis in HIV infection: the relationship of the radiographic appearance to CD4 t-lymphocytes count. Malays J Med Sci.

[14] Tiamiyu AB, Iliyasu G, Dayyab FM (2020). A descriptive study of smear negative pulmonary tuberculosis in a high HIV burden patient's population in North Central Nigeria. PLoS One.

[15] Leeds IL, Magee MJ, Kurbatova EV, del Rio C, Blumberg HM, Leonard MK, Kraft CS (2012). Site of extrapulmonary tuberculosis is associated with HIV infection. Clin Infect Dis.

[16] Bjerrum S, Schiller I, Dendukuri N (2019). Lateral flow urine lipoarabinomannan assay for detecting active tuberculosis in people living with HIV. Cochrane Database Syst Rev.

[17] Takhar RP, Mirdha K, Purohit G, Maan L, Bainara MK (2018). Impact of HIV co-infection on clinical presentation in patients with tb and correlation of the findings with level of immune suppression. Tanaffos.

[18] (2024). CDC: Clinical Testing Guidance for HIV. Clin.

[19] Davis JL, Huang L, Worodria W (2011). Nucleic acid amplification tests for diagnosis of smear-negative TB in a high HIV-prevalence setting: a prospective cohort study. PLoS One.

[20] Kim HN, Lee J, Yoon SY, Jang WS, Lim CS (2023). Rapid detection of mycobacterium tuberculosis using a novel point-of-care BZ TB/NTM NALF assay: integrating LAMP and LFIA technologies. Diagnostics (Basel).

[21] Donovan J, Thu DD, Phu NH (2020). Xpert MTB/RIF Ultra versus Xpert MTB/RIF for the diagnosis of tuberculous meningitis: a prospective, randomised, diagnostic accuracy study. Lancet Infect Dis.

[22] Gupta-Wright A, Manabe YC (2019). Implementation science: point-of-care diagnostics in HIV and tuberculosis. Clin Med (Lond).

[23] Matee M, Mtei L, Lounasvaara T (2008). Sputum microscopy for the diagnosis of HIV-associated pulmonary tuberculosis in Tanzania. BMC Public Health.

[24] Mase SR, Chorba T (2019). Treatment of drug-resistant tuberculosis. Clin Chest Med.

[25] Bekker L-G, Wood R (2011). TB and HIV co-infection: when to start antiretroviral therapy. Contin Med Educ.

[26] Ramappa V, Aithal GP (2013). Hepatotoxicity related to anti-tuberculosis drugs: mechanisms and management. J Clin Exp Hepatol.

[27] Eggleton JS, Nagalli S (2024). Highly active antiretroviral therapy (HAART). StatPearls [Internet].

[28] (2008). Implementing the WHO Stop TB Strategy: A Handbook for National Tuberculosis Control Programmes. World Health Organization.

[29] Gengiah TN, Gray AL, Naidoo K, Karim QA (2011). Initiating antiretrovirals during tuberculosis treatment: a drug safety review. Expert Opin Drug Saf.

[30] Moore BK, Graham SM, Nandakumar S, Doyle J, Maloney SA (2024). Pediatric tuberculosis: a review of evidence-based best practices for clinicians and health care providers. Pathogens.

[31] Zhou X, Fang G, Xie Y, Wei A, Huang F (2022). Safety evaluation of antituberculosis drugs during pregnancy: a systematic review and meta-analysis. Front Surg.

[32] Cohen K, Meintjes G (2010). Management of individuals requiring antiretroviral therapy and TB treatment. Curr Opin HIV AIDS.

[33] Dersch-Mills D (2018). Assessment considerations in pediatric patients. Patient Assess Clin Pharm.

[34] Yanes-Lane M, Ortiz-Brizuela E, Campbell JR, Benedetti A, Churchyard G, Oxlade O, Menzies D (2021). Tuberculosis preventive therapy for people living with HIV: a systematic review and network meta-analysis. PLoS Med.

[35] González Fernández L, Casas EC, Singh S (2020). New opportunities in tuberculosis prevention: implications for people living with HIV. J Int AIDS Soc.

[36] Tait DR, Hatherill M, Van Der Meeren O (2019). Final analysis of a trial of M72/AS01(E) vaccine to prevent tuberculosis. N Engl J Med.

[37] Sculier D, Getahun H, Lienhardt C (2011). Improving the prevention, diagnosis and treatment of TB among people living with HIV: the role of operational research. J Int AIDS Soc.

[38] Dean HD, Fenton KA (2010). Addressing social determinants of health in the prevention and control of HIV/AIDS, viral hepatitis, sexually transmitted infections, and tuberculosis. Public Health Rep.

[39] Pathmanathan I, Ahmedov S, Pevzner E, Anyalechi G, Modi S, Kirking H, Cavanaugh JS (2018). TB preventive therapy for people living with HIV: key considerations for scale-up in resource-limited settings. Int J Tuberc Lung Dis.

[40] Dlatu N, Oladimeji KE, Apalata T (2023). Voices from the patients: a qualitative study of the integration of tuberculosis, human immunodeficiency virus and primary healthcare services in O.R. Tambo district, Eastern Cape, South Africa. Infect Dis Rep.

[41] Kalonji D, Mahomed OH (2019). Health system challenges affecting HIV and tuberculosis integration at primary healthcare clinics in Durban, South Africa. Afr J Prim Health Care Fam Med.

[42] Hargreaves JR, Boccia D, Evans CA, Adato M, Petticrew M, Porter JD (2011). The social determinants of tuberculosis: from evidence to action. Am J Public Health.

[43] George LS, Rakesh PS, Vijayakumar K, Kunoor A, Kumar A (2020). Social stigma associated with TB and HIV/AIDS among Kudumbashree members: a crosssectional study. J Family Med Prim Care.

[44] Spooner E, Reddy S, Ntoyanto S (2022). TB testing in HIV-positive patients prior to antiretroviral treatment. Int J Tuberc Lung Dis.

[45] Research C for DE and: New Drug Therapy Approvals 2022. FDA. (2024). Research C for DE and: New Drug Therapy Approvals 2022. February 3, 2024.

[46] Dooley KE, Nuermberger EL, Diacon AH (2013). Pipeline of drugs for related diseases: tuberculosis. Curr Opin HIV AIDS.

[47] Lawn SD (2015). Advances in diagnostic assays for tuberculosis. Cold Spring Harb Perspect Med.

[48] (2024). WHO announces updates on new TB antigen-based skin tests for the diagnosis of TB infection. https://www.who.int/news/item/04-04-2022-who-announces-updates-on-new-tb-antigen-based-skin-tests-for-the-diagnosis-of-tb-infection.

[49] (2024). WHO announces updates to its guidelines on tests for the diagnosis of TB infection. https://www.who.int/news/item/30-09-2022-who-announces-updates-to-its-guidelines-on-tests-for-the-diagnosis-of-tb-infection..

[50] Venkatesan P (2022). Worrying lack of funding for tuberculosis. Lancet Infect Dis.

[51] Deshkar AT, Shirure PA (2022). Bedaquiline: a novel diarylquinoline for multidrug-resistant pulmonary tuberculosis. Cureus.

[52] Xavier AS, Lakshmanan M (2014). Delamanid: a new armor in combating drug-resistant tuberculosis. J Pharmacol Pharmacother.

[53] Seung KJ, Keshavjee S, Rich ML (2015). Multidrug-resistant tuberculosis and extensively drug-resistant tuberculosis. Cold Spring Harb Perspect Med.

[54] Patel PH, Hashmi MF (2024). Macrolides. StatPearls [Internet].

